# Experimental Evaluation of 6BLEMesh: IPv6-Based BLE Mesh Networks

**DOI:** 10.3390/s20164623

**Published:** 2020-08-17

**Authors:** Seyed Mahdi Darroudi, Carles Gomez

**Affiliations:** Department of Network Engineering, Universitat Politècnica de Catalunya, C/Esteve Terradas, 7, 08860 Castelldefels, Spain; carlesgo@entel.upc.edu

**Keywords:** bluetooth low energy, BLE, mesh, 6LoWPAN, 6Lo, experimental evaluation, energy modeling, internet of things, IoT

## Abstract

Bluetooth Low Energy (BLE) has become a major wireless technology for the Internet of Things (IoT). Recent efforts of academia, industry and standards development organizations have focused on creating BLE mesh network solutions. 6BLEMesh is a specification being developed by the IETF that defines an IPv6-oriented approach for BLE mesh networking. In this paper, we perform an experimental evaluation of 6BLEMesh, based on a real implementation. We evaluate latency, round trip time (RTT) and energy consumption. For the latter, we model the device current consumption, we determine the energy efficiency of communication, and we obtain the theoretical device lifetime (for battery-operated devices), for three different hardware platforms. Under the assumptions in our study (including a simple 235 mAh battery, and periodic data transmission), the maximum, asymptotic, device lifetime is 573 days, whereas battery-operated router devices can also achieve 3-digit lifetimes (in days) in many scenarios. Our results also illustrate the impact on performance of BLE-level and application-level parameter settings, adaptation layer mechanisms such as IPv6 header compression, and device hardware characteristics.

## 1. Introduction

Bluetooth Low Energy (BLE) is a wireless communications technology created by the Bluetooth Special Interest Group (SIG), and introduced as part of the Bluetooth 4.0 core specification [1]. In contrast with previous Bluetooth versions, BLE is mainly intended for applications involving simple devices (e.g., battery-enabled sensors) that need to infrequently transfer small units of data with remarkably low power consumption. Thanks to these characteristics and fueled by its common support in consumer electronics devices (e.g., smartphones), BLE has become a major communications technology for the Internet of Things (IoT). The interest of industry in BLE has led to several updates of the BLE core specifications (Bluetooth 5.2 being currently the most recent one) [2,3,4,5,6].

Originally, BLE only supported the star network topology. However, many competing technologies in the IoT space support the mesh network topology as well, which provides advantages in terms of robustness and covered area. While Bluetooth 4.1 removed the constraints that only allowed the star topology in BLE, no subsequent Bluetooth core specification defined how to enable end-to-end communication on a mesh topology. In parallel, many academic and proprietary proposals were designed in order to cope with this limitation [7].

Industry and academic interest in BLE mesh networks triggered two standardization initiatives intended to enable BLE mesh networks, called Bluetooth Mesh [8,9,10] and IPv6-based BLE mesh networks (6BLEMesh) [11], produced by the Bluetooth SIG and the IETF, respectively. Bluetooth Mesh and 6BLEMesh follow different technical approaches [12]. A distinctive 6BLEMesh feature is that, in contrast with Bluetooth Mesh, 6BLEMesh supports IPv6, which allows seamless Internet integration. 6BLEMesh is currently in the final stages of the IETF process towards publication of its specification. Therefore, 6BLEMesh is currently mature enough to focus on understanding its performance characteristics.

In this paper, and to the best of our knowledge, we provide the first experimental evaluation of 6BLEMesh. We focus on latency, round trip time (RTT), and energy consumption. For the latter, we measure the performance differences of using different device hardware platforms, and develop models to predict crucial parameters, such as lifetime of battery-operated devices and energy efficiency of communication. In the conditions assumed in our study (including the use of a simple 235 mAh battery, and periodic data transmission), the maximum device lifetime found is 573 days. Our results also illustrate the impact on performance of BLE-level and application-level parameter settings, along with device hardware characteristics.

The remainder of this paper is organized as follows: Section 2 overviews the main technical background concepts for 6BLEMesh. Section 3 reviews related work. Section 4 introduces the testbed that we have used to make experimental evaluations. Section 5 provides 6BLEMesh latency and RTT measurement results. Section 6 presents 6BLEMesh energy consumption modeling and evaluation results. Section 7 investigates the feasibility of battery-operated 6LRs. Finally, Section 8 concludes the paper.

## 2. Technical Background

6BLEMesh specifies an IPv6-based solution for end-to-end communication in a BLE mesh network topology, by adapting IPv6 over Low-Power Wireless Personal Area Networks (6LoWPANs) [13]. This section introduces the main technical concepts in 6BLEMesh. The section is organized into three subsections. The first two subsections overview BLE and 6LoWPAN, respectively, emphasizing the main components that are used in 6BLEMesh. The third subsection describes 6BLEMesh.

### 2.1. BLE

**Figure 1:**
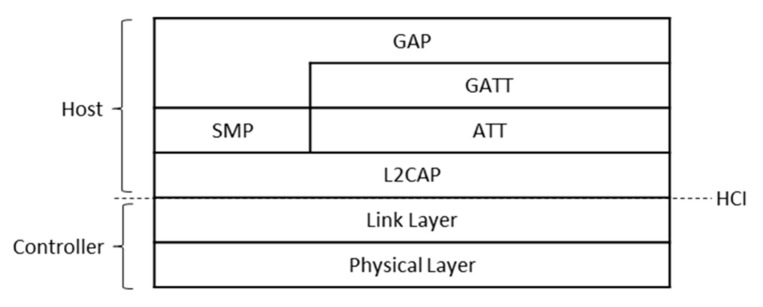
Bluetooth Low Energy protocol stack.

BLE is defined as a full protocol stack (see Figure 1). The BLE protocol stack comprises two main sections: the Controller and the Host, which are communicated via the Host Controller Interface (HCI). The Controller handles lower layer (including hardware) responsibilities, while the Host performs upper layer tasks. The former comprises the Physical Layer and the Link Layer, while the latter is composed of the Logical Link Control and Adaptation Protocol (L2CAP), the Attribute protocol (ATT), the Generic Attribute profile (GATT), the Security Manager Protocol (SMP) and Generic Access Profile (GAP) layers.

The BLE Physical Layer defines 40 different frequency channels in the 2.4 GHz Industrial Scientific Medical (ISM) band. The channels are divided into three advertising channels and 37 data channels. The Physical Layer bit rate in Bluetooth 4.x specifications is 1 Mbit/s (other bit rates, ranging from 125 kbit/s to 2 Mbit/s were added in Bluetooth 5.0).

**Figure 2:**
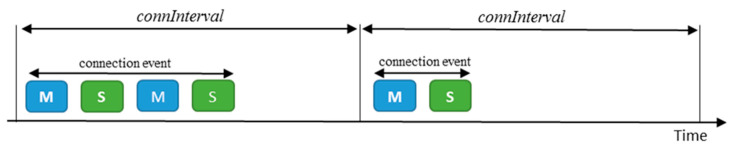
Examples of connection events (and their corresponding BLE packet exchanges) between two connected BLE devices. One of the endpoints of a connection runs as a Master (M), whereas the other one plays the role of a slave (S). The sender of a BLE packet is indicated as M or S in the figure.

Two different communication paradigms are supported in BLE. The first one is unidirectional message transmission, which uses the advertising channels. The second paradigm is bidirectional data transmission, which makes use of data channels between neighboring devices that have established a Link Layer connection. In a connection, one endpoint acts as a master, while the other plays the role of a slave. Every *connInterval* time, the master sends a packet to the slave, initiating a packet exchange between both endpoints called connection event (see Figure 2). Once the connection event is closed, the slave may remain in sleep mode to save energy, until the start of the next connection event. The master manages the connection and handles simultaneous connections with different slaves by means of a Time Division Multiple Access (TDMA) scheme.

The L2CAP supports several services, such as segmentation and reassembly of upper layer data units, flow control and reliable transmission. Upper layer functionality comprises application-layer interactions between a client and a server, service discovery and device configuration. However, the layers on top of the L2CAP are not used in 6BLEMesh, and therefore are out of the scope of this paper.

### 2.2. 6LoWPAN

6LoWPAN is a protocol suite designed to support IPv6 over IEEE 802.15.4 networks [13]. Like BLE networks, IEEE 802.15.4 networks typically consist of devices that are constrained in terms of computation, energy, and communication resources. Since IPv6 was created for significantly less restricted networks, 6LoWPAN adapts IPv6 operation for constrained networking environments.

6LoWPAN offers three basic components: (1) fragmentation, (2) IPv6 and UDP header compression, and (3) IPv6 Neighbor Discovery (ND) optimization. The first mechanism allows complying with the IPv6 requirement that packets of at least 1280 bytes need to be supported by the layer below IPv6; note that maximum frame payload sizes in IEEE 802.15.4 are typically up to only ~100 bytes. The last two mechanisms enable lightweight operation in resource-constrained environments. Header compression in 6LoWPAN produces efficient header encoding prior to packet transmission, whereas 6LoWPAN-optimized ND minimizes multicast operation and allows energy-constrained devices to remain most of the time in a low energy consumption state.

IEEE 802.15.4 supports the mesh topology. Accordingly, 6LoWPAN defines three node types to handle the described operation in such topology:

- 6LoWPAN Border Router (6LBR): it is an edge router that connects the 6LoWPAN network with the Internet. It often supports several network interfaces, including one used for communication with the 6LoWPAN network, and at least another one that offers Internet connectivity (e.g., Wi-Fi, 4G, etc.).
- 6LoWPAN Node (6LN): it is an end-device, without routing capabilities. 6LNs are typically simple devices with restricted energy sources (e.g., small batteries). They may be directly connected to a 6LR or a 6LBR.
- 6LoWPAN Router (6LR): it is an internal 6LoWPAN network router. It typically relays packets originated at, or intended for, a 6LN.

### 2.3. 6BLEMesh

In 2015, the IETF published RFC 7668, which specifies IPv6 over BLE networks by leveraging 6LoWPAN functionality [14]. However, RFC 7668 just supports star topology BLE networks. In such topology, there is a central node that plays the BLE master role and acts as a 6LBR, that handles simultaneous Link Layer connections with one-hop BLE slaves acting as 6LNs. Communication occurs over data channels via the established connections. The adaptation layer defined by RFC 7668 is located between IPv6 and the L2CAP layer of BLE, since this layer provides segmentation and reassembly, and therefore fragmentation functionality at the 6LoWPAN level is not needed.

In order to expand the range of supported BLE topologies, the IETF IPv6 over Networks of Resource-constrained nodes (6Lo) working group has been developing a new standard, called 6BLEMesh, which aims to carry IPv6 packets over BLE networks that use the mesh topology [11]. To this end, 6BLEMesh adds the following functionality to RFC 7668:

- 6BLEMesh restores the 6LR role for intermediate routers in multihop topologies.
- 6BLEMesh restores also the multihop functionality supported in 6LoWPAN-optimized ND, including Duplicate Address Detection (DAD) centralized at the 6LBR.
- A modified header compression mechanism is specified in 6BLEMesh. RFC 7668 fully omitted the source IPv6 address of a packet sent by a 6LN, since the 6LBR knows the source of packets received via a given BLE connection. Similarly, the destination IPv6 address packets sent to a 6LN was omitted as well. However, since 6BLEMesh targets a multihop scenario, such optimization can only be applied in the first/last link, i.e. a link where a 6LN is an endpoint.
- For end-to-end communication, 6BLEMesh requires a route-over routing solution, which is not specified and can thus be selected by implementers accordingly to scenario requirements.

Note that, in 6BLEMesh, devices may be more complex than RFC 7668 ones, since in 6BLEMesh a device may run both the master and the slave roles simultaneously.

## 3. Related Work

This section overviews the literature in the topics related with this paper, that is, BLE mesh networking, with a focus on performance evaluation of standardized BLE mesh network solutions, including 6BLEMesh.

The section is organized in three subsections. The first one introduces and discusses the main categories of BLE mesh networking proposals. The second subsection reviews Bluetooth Mesh studies. The last subsection discusses work related with or influenced by 6BLEMesh. Note that, since 6BLEMesh is a novel standardized solution, still being completed as of the writing, the body of related literature is still narrow, currently.

### 3.1. BLE Mesh Network Proposals

Before the publication of the Bluetooth Mesh standard, authors in [7] collected existing BLE mesh networking proposals and created a taxonomy for BLE mesh network solutions. Solutions were classified into academic solutions, proprietary solutions, and standardized solutions (the latter containing only standards that were being developed at the time). Academic solutions were divided into flooding-based [15,16] and connection-based solutions [17,18,19,20,21,22,23,24], with the latter being further classified based on their routing approach into static routing [17,18] or dynamic routing [19,20,21,22,23,24]. Proprietary solutions were analyzed as well [25,26,27,28,29,30,31,32,33,34].

Academic, non-standards-based, BLE mesh networking solutions have recently been created as well [35,36,37]. However, increasing interest in BLE mesh networks triggered two standardization initiatives intended to enable communication between devices in BLE mesh networks: Bluetooth Mesh (produced by the Bluetooth SIG), and 6BLEMesh (developed by the IETF). Bluetooth Mesh uses managed flooding over advertising channels, which is fundamentally different from the end-to-end delivery mechanism used in 6BLEMesh.

Bluetooth Mesh and 6BLEMesh were comprehensively investigated and compared in [12]. The work evaluated the following performance parameters and characteristics of the two considered BLE mesh networking standards: message transmission count, link corruption robustness, and variable topology robustness, latency, energy consumption, protocol encapsulation overhead and Internet connectivity.

### 3.2. Bluetooth Mesh

While Bluetooth Mesh is a relatively recent standard, several published studies have already evaluated it in terms of different performance parameters.

One of the first Bluetooth Mesh standard evaluation studies focused on latency performance [38]. Its authors showed the impact of network density on latency, and how a backoff mechanism can increase the network reliability and scalability, at the expense of increased latency.

The authors in [39] provided a model to predict critical energy consumption parameters of Low Power Nodes (LPN) in Bluetooth Mesh. They developed an analytical current consumption model based on experimental measurements on real hardware. They used the model to determine LPN average current consumption, node lifetime and energy cost per delivered bit.

Another work evaluated analytically the effect of different network settings on network reliability, delay and scalability [40]. The authors also simulated Bluetooth Mesh functionality in the presence of WLAN interference. They concluded that Bluetooth Mesh does not take into consideration the effect of self-interference, while external interference strongly affects Bluetooth Mesh performance.

A smart office use case was proposed in [41], where the authors deployed Bluetooth Mesh nodes in a building. The setup covered all roles defined in the Bluetooth Mesh specification in their developed topology. The nRF52 SoC from Nordic Semiconductor was used for LPNs, while Raspberry Pi 3 Model B with the Linux operating system was used for grid-powered nodes. Authors analyzed the characteristics of the flooding approach used in Bluetooth Mesh, with a considerable number of duplicate packets, and a significant forwarding activity by relay nodes.

The authors in [42] challenged the friendship role in Bluetooth Mesh from a security point of view. They identified vulnerabilities that may be leveraged for impersonation or denial-of-service attacks. Moreover, they devised a tool, namely btlemesh, which is able to analyze the security level of a specific Bluetooth Mesh node.

In [43] the authors investigated possible parameter settings throughout the whole Bluetooth Mesh protocol stack. They reported configuration challenges and outlined some areas for improvement of the standard.

The feasibility of using Bluetooth Mesh for indoor localization systems has also been studied [44]. The authors experimentally evaluated Bluetooth Mesh functionality under different localization algorithms and found that Bluetooth Mesh offers similar performance in comparison with other competitive technologies by using algorithms that generate a lower amount of data traffic. Another indoor case study on Bluetooth Mesh evaluated the impact of interference on network performance by comparing evaluation results in working and non-working days [45].

Finally, a smart home solution based on Bluetooth Mesh has been developed [46], by using Nordic nrf52832 devices. The authors implemented a Bluetooth Mesh prototype that collects sensor data from distributed nodes and transfers the data to a central database node.

### 3.3. IPv6-Based BLE Mesh Networking

There exist few research works on IPv6-based BLE mesh networking, either focusing on 6BLEMesh or following an independent approach.

Use of a parametrization layer was introduced by the authors of the BLEach implementation of IPv6 over BLE [47]. They argued that such a layer can act as a building block to create IPv6 over BLE mesh networks using connectionless BLE. Note that 6BLEMesh follows a connection-based approach.

The authors in [48] experimentally studied a connected IPv6-based BLE mesh three-node topology, in terms of energy consumption, delivery rate and goodput. They developed a prototype by using a Texas Instruments CC1350 and focused on three-node topologies. Nodes ran a modification of BLEach on Contiki OS [49]. However, their implementation was not compliant with the 6BLEMesh specification.

Another approach to support IPv6-based BLE mesh networking was based on emulating a 6LoWPAN network (including 6LBR, 6LR and 6LN roles) on top of Bluetooth Mesh [50] IPv6 functionality, such as address generation, was carried out at the application layer in this work.

A new Neighbor Discovery (ND) approach for 6BLEMesh was proposed in [51]. Authors extended the 6LoWPAN ND defined in RFC 7668 [14] to support multi-hop topologies, along with duplicate address detection. Authors evaluated their proposed mechanism analytically and by simulation, in terms of probability of successful discovery, average discovery latency, and average energy consumption of an advertiser during the ND process.

As of the writing of this article, to our best knowledge, no prior published work provides an experimental evaluation of 6BLEMesh.

## 4. Testbed Details

**Figure 3:**
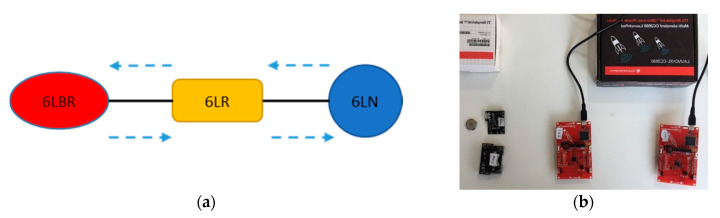
Prototype testbed of the 6BLEMesh implementation: (**a**) the topology; (**b**) device platforms.

In order to evaluate 6BLEMesh experimentally, we developed a three-node prototype testbed that comprises a 6LN, a 6LR, and a 6LBR (Figure 3a). Therefore, this prototype testbed comprises all 6LoWPAN node roles. Note that there exist real use cases based on small scale deployments, especially in the early stages of real world IoT adoption. For example, in the remote healthcare domain, an on-body medical sensor (i.e., a 6LN) may transmit data towards a home router (i.e., a 6LBR) via an intermediate device (a 6LR) that is needed to enable end-to-end connectivity between the 6LN and the 6LBR.

On the other hand, the experiments performed by using this testbed may also represent a reference to understand or estimate performance results in more complex scenarios (see Section 7).

We implemented the different roles on Texas Instruments CC2650 platforms (Dallas, TX, USA) [52], including CC2650 LaunchPad [53] (in short, LaunchPad), and CC2650 SensorTag [54] (in short, SensorTag). The 6LN and the 6LR run on a LaunchPad, while the 6LBR runs on a SensorTag.

Figure 3b shows a picture of the devices used in our testbed. In the evaluations, neighboring nodes were separated at a distance of 2 meters from each other, Bluetooth 4.1 was used, and the transmit power was set to 0 dBm. For better control over the testbed topology, we used static routing. We used a packet size carrying an 18-byte user payload, which does not exceed 27 bytes at the BLE Link Layer, and it fits into one Physical Layer data unit.

We implemented 6BLEMesh by modifying and extending an RFC 7668-compliant implementation called BLEach [47], which runs on top of Contiki (city, country). Contiki is a lightweight operating system that aims to be used by resource-restricted nodes (e.g., sensor nodes) [49]. As a side-contribution of this paper, we publicly offer our 6BLEMesh implementation code [55].

## 5. Delay Measurements

We conduct two types of delay-related evaluations on the testbed described in Section 4. First, we measure one-hop, one-way, latency of packet transmission. Secondly, we study round trip time (RTT). This section is divided into two subsections, which focus on each one of the performance parameters mentioned, respectively.

### 5.1. One-Hop, One-Way Latency

We measure one-hop latency as the total time required for sending an IPv6 packet to a sender’s one-hop neighbor. We consider two cases: (1) the sender is also the packet source, and (2) the sender just relays the packet. In the first case, we study the latency of packet transmission from the 6LN to the 6LR. In the second one, we evaluate the latency of packet transmission between the 6LR and the 6LBR.

From an analytical point of view, the IPv6 packet may be sent at any time during the current connection interval, of *connInterval* duration. Thus, the expected time to actually send an IPv6 packet from the sender node is *connInterval*/2, plus the packet transmission time. However, if occasionally the IPv6 packet cannot be delivered during the current connection interval, it will be delivered in subsequent connection intervals.

**Figure 4:**
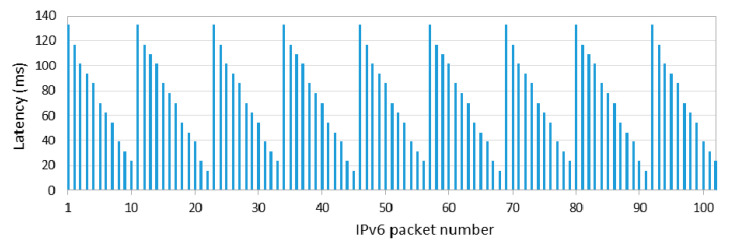
One-hop IPv6 packet delivery latency when the sender (the 6LN in our testbed) is also the packet source (for *connInterval* = 125 ms).

**Figure 5:**
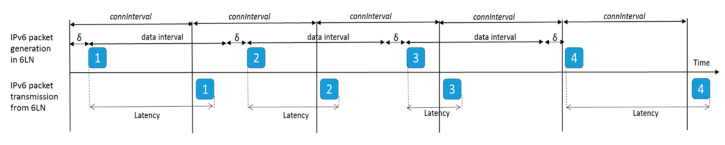
Time diagram for an example that illustrates one-hop IPv6 packet latency when the sender (the 6LN in our testbed) is also the packet source (for *connInterval* = data interval). In the example, four packets are transmitted (the packets are identified by numbers 1, 2, 3 and 4).

Figure 4 shows one-hop, one-way latency when the sender (the 6LN in Figure 3a) is also the IPv6 packet source, with the 6LR as a receiver, for 1000 consecutive IPv6 packets, with an inter-packet time of data interval = 1 s, and *connInterval* set to 125 ms. The measured latency shown in Figure 4, and further illustrated in Figure 5, is defined as the time since the packet has been generated and is ready for transmission at the sender until the time in which the packet transmission is complete. As shown in Figure 4, the first packet cannot be sent in the current connection interval, and it is actually sent at the beginning of the second one, after 132.813 ms. This time is equal to *connInterval* plus the Contiki tick clock time of the 6LN in our testbed, which is 7.813 ms (i.e., the inverse of 128 Hz). For each subsequent packet sent, latency decreases from the previous packet latency by a delay, denoted *δ*, of 1–2 tick clock periods, as the actual packet generation and handling is delayed by that time. The time since packet generation until the next connection event decreases steadily for a cycle of 11–12 packets, until a packet needs to wait again for a full *connInterval* for the next connection event. The described behavior repeats again thereafter, leading to the sawtooth shape shown in Figure 4.

Figure 5 illustrates further the reasons for the described latency behavior shown in Figure 4, based on an example that, for the sake of clarity, is based on simplified assumptions. In the example, data interval is equal to *connInterval*, and a cycle only comprises three packets. As shown in Figure 5, the latency between packet generation and complete packet transmission decreases for packets 1, 2 and 3 (due to the additional delay *δ*). However, packet 4 is generated too late to be sent at the beginning of its current *connInterval*, thus it is sent at the beginning of the subsequent *connInterval*, increasing latency again and starting a new cycle.

**Figure 6:**
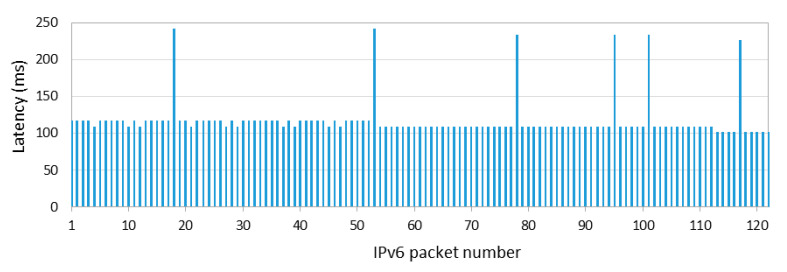
One-hop IPv6 packet delivery latency when the sender (the 6LR in our testbed) only relays the packet received from the 6LN to the 6LBR (for *connInterval* = 125 ms).

**Figure 7:**
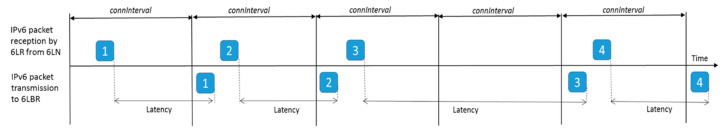
Time diagram for an example that illustrates one-hop IPv6 packet latency when the sender (the 6LR in our testbed) only relays the packets received from the 6LN to the 6LBR. In the example, four packets are transmitted (the packets are identified by numbers 1, 2, 3 and 4).

The previous study was carried out on a network where the sender was also the IPv6 packet source. We next also study the one-way packet transmission latency between two neighboring nodes where the sender is not the IPv6 packet source; instead, the sender (the 6LR in Figure 3a) only receives the IPv6 packet from a previous node, and relays the IPv6 packet to the next node (the 6LBR). Figure 6 illustrates the obtained latency results, for the same *connInterval* setting (i.e., 125 ms) as in the previous experiment. The measured latency shown in Figure 6, and further illustrated in Figure 7, is defined as the time since the packet has been received by the 6LR, and thus is ready for transmission by the latter, until the time in which the packet transmission by the 6LR is complete. There are three remarkable observations from Figure 6. First, most IPv6 packets are delivered at the beginning of the next connection interval (of the connection between the 6LR and the 6LBR), leading to a latency of slightly less than 125 ms (by amounts of time that are a multiple of 7.813 ms, i.e., the Contiki tick clock). Note that the 6LR receives packets from its connection with the 6LN, but the 6LR cannot deliver those packets immediately via its connection with the 6LBR. Secondly, there are a few IPv6 packets that are not delivered to the 6LBR in the next connection interval, and are therefore transmitted during the subsequent one, duplicating one-hop packet latency. Thirdly, latency is quite constant in Figure 6 since the 6LR does not need to generate the IPv6 packet. Thus, the received IPv6 packet is typically sent in the same position of the corresponding subsequent connection interval. Further details for the three provided observations are illustrated in Figure 7, which shows a time diagram for the reception from the 6LN and transmission by the 6LR of four packets.

We made additional experiments for other *connInterval* settings, finding similar behaviors as the ones observed in Figure 4 and Figure 6, relative to each corresponding *connInterval* setting.

### 5.2. Round Trip Time

We now measure the RTT on the two-hop topology depicted in Figure 3a. We run an application on the 6LN that periodically sends a message intended for the 6LBR. The 6LR relays packets received from the 6LN to the 6LBR. While the 6LBR would typically route the corresponding IPv6 packet towards the Internet, for our measurement purposes we set the 6LBR to return such messages back to the 6LR immediately. The 6LR forwards the packets received from the 6LBR back to the 6LN. We have logged the sending time of each packet from the 6LN and the reception time of the same packet by the 6LN in order to determine the corresponding RTT.

**Figure 8:**
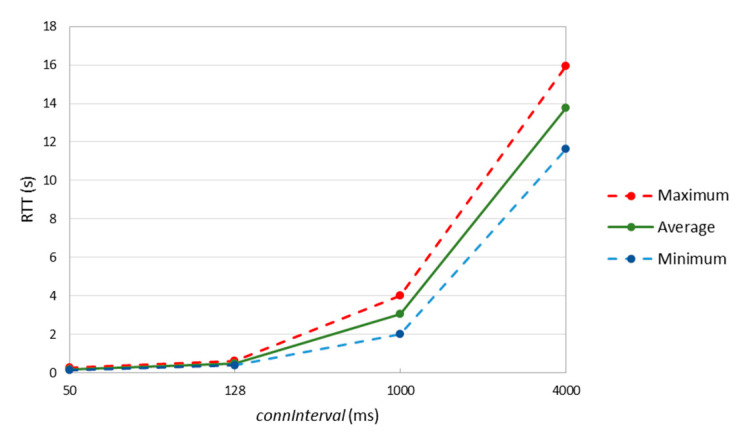
Measured IPv6 packet RTT results over a two-hop topology.

Figure 8 shows the main RTT statistics obtained as described, based on 1000 individual packet transmissions (performed every 1 s), for various *connInterval* settings from 50 ms to 4 s. *connInterval* values lower than 50 ms did not allow stable operation for the devices used in the experiments.

Results show that, when *connInterval* is set to 1 s, the minimum, average, and maximum measured RTT values are approximately equal to 2, 3 and 4 s, respectively. These results correspond to 2, 3 and 4 *connInterval* periods, respectively, which is consistent with the results in Section 5.1, by which one-hop and one-way latency results can take values from almost zero up to *connInterval* (when the sender is the packet source), and typically equal to *connInterval* (when the sender is a relay). However, for *connInterval* of 4 s, the packet sending interval (of 1 s) is lower than *connInterval*. For this setting, the minimum and the average RTT latency increase, which we attribute to node queuing delay. An additional comment is that we observed packet drops, in general, when the time between consecutive packets exceeded *connInterval*, due to memory overflow events.

## 6. Energy Consumption Modeling

As introduced in earlier sections, there are three node roles in 6BLEMesh: the 6LN, the 6LR, and the 6LBR. The last two, being routers, are better suited for devices with a good energy supply (e.g., the electricity grid). However, the 6LN is expected to be an energy-constrained device, e.g., running on a simple battery as its energy source.

In this section, we model the 6LN current consumption, and we evaluate important energy-related performance parameters.

First, we study the current consumption of a 6LN in two situations: (1) in a connection interval without actual user data transmission (i.e., with an empty packet exchange that only keeps the BLE connection alive), and (2) in a connection interval wherein the device transmits one IPv6 data packet (hereafter, called data packet) every connection interval. These measurements allow us to derive a 6LN current consumption model. Then, we study the energy efficiency that corresponds to different data packet transmission intervals, and the device lifetime of the considered device types for different data transmission intervals. Finally, we evaluate the energy performance improvement of 6BLEMesh IPv6 header compression.

Since different hardware platforms exhibit different current consumption characteristics, our model is created, and evaluations are performed, considering three popular commercial BLE device platforms: (1) Nordic Semiconductor PCA10028 (Trondheim, Norway) [56] (in short, PCA10028), (2) LaunchPad, and (3) SensorTag. In the section, we measure the current consumption of the aforementioned devices by using an Agilent N6705A Power Analyzer (Santa Clara, CA, USA) [57].

### 6.1. Current Consumption during a Connection Interval without Data Transmission

The 6LN will often participate in connections where not all connection intervals include the exchange of BLE data units carrying IPv6 packets. Note that, to maintain link-level connectivity and synchronization, both endpoints of a BLE connection will always exchange BLE data units every *connInterval*. Accordingly, we have measured the current consumption of connection intervals without actual data transmission.

**Figure 9:**
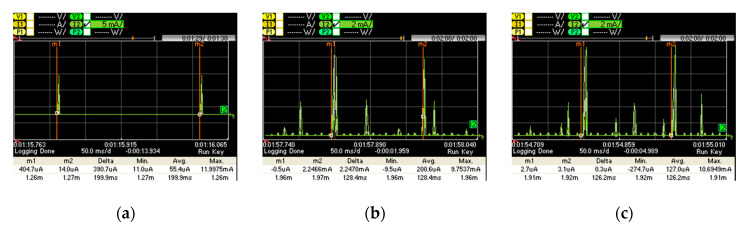
Current consumption of one connection interval without data transmission: (**a**) PCA10028; (**b**) LaunchPad; (**c**) SensorTag.

Figure 9 shows the current consumption of a connection event with no data packet transmission for (a) PCA10028, (b) LaunchPad, and (c) SensorTag. We perform current consumption measurements for the device in the slave role, as the master role is expected for its connection endpoint (a 6LR or a 6LBR). The slave is most of the time in sleep mode. When a new connection event starts, the slave wakes up, it receives one packet from the master, it replies by sending another packet to the master, and it returns to sleep mode.

**Figure 10:**
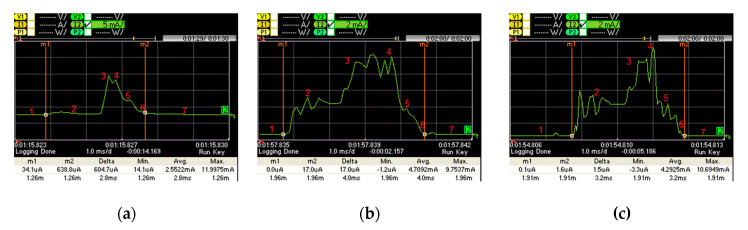
Current consumption of the active part of a connection interval without data transmission: (**a**) PCA10028; (**b**) LaunchPad; (**c**) SensorTag.

Figure 10 illustrates the active part of the connection interval shown in Figure 9. We identified 7 current consumption states, namely: sleep mode, wake up, packet reception from the master, packet response to the master, post-processing, radio off and cool down.

Table 1 provides the current consumption and time characteristics of each state. Figure 10 shows how the hardware platforms considered follow different approaches that exhibit different current consumption patterns, due to BLE implementation differences.

Based on the current consumption characterization shown in Table 1, it is possible to determine the average current consumption of a slave device in a BLE connection without actual data packet transmission (denoted *I_NoData-CI_*). This variable can be obtained by using Equation (1):

(1) $$I_{NoData-CI}=\left(\overset{7}{\underset{i=1}{\sum }}T_{i}\cdot I_{i}\right)$$

All values of *T_i_* and *I_i_* represent the state durations and corresponding current consumptions shown in Table 1, except for the sleep duration (*T*_1_ which is denoted *T_Sleep-ND_*). The latter can be computed by using Equations (2) and (3), where *T_CI_* refers to *connInterval* and *T_ND-active_* denotes the sum of the active time parts within *connInterval*:

(2) $$T_{Sleep-ND=\;}T_{CI\;}-\;T_{ND-active}$$

(3) $$T_{ND-active=\;}\;\left(\overset{7}{\underset{i=2}{\sum }}T_{i}\right)$$

**Figure 11:**
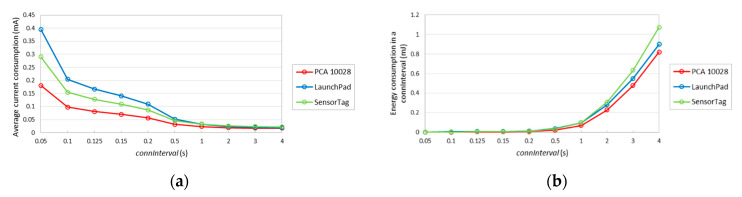
(**a**) Average current consumption for different *connInterval* settings, without data packet transmission; (**b**) energy consumption during a connection interval, without data packet transmission, as a function of *connInterval*.

The average current consumption of a slave, without data packet transmission, for the different devices considered, and for different *connInterval* settings, is depicted in Figure 11a. As expected, the average current consumption decreases with *connInterval*, since the influence of sleep intervals increases. For example, when *connInterval* is set to 50 ms, the average current consumptions of PCA10028, LaunchPad and SensorTag are 0.181, 0.395 and 0.290 mA, respectively, whereas for *connInterval* equal to 4 s, the respective average consumptions are 0.017, 0.018 and 0.022 mA. Note that, in state 2 (see Table 1), during its wake-up process, PCA10028 consumes significantly less current than LaunchPad and SensorTag, while state 3 is shorter for PCA10028. In consequence, the average current consumption by PCA10028 is lower than that of the other considered devices.

Figure 11b depicts the energy consumption of each device type in the slave role, during one complete connection interval, without data packet transmission. For *connInterval* lower than 1 s, LaunchPad consumes more energy in one *connInterval*, while otherwise SensorTag consumes a greater amount of energy in one *connInterval*. This happens because the current consumption during sleep mode in SensorTag is higher than the LaunchPad one.

### 6.2. Current Consumption during a ConnectionIinterval, with Data Packet Transmission

**Figure 12:**
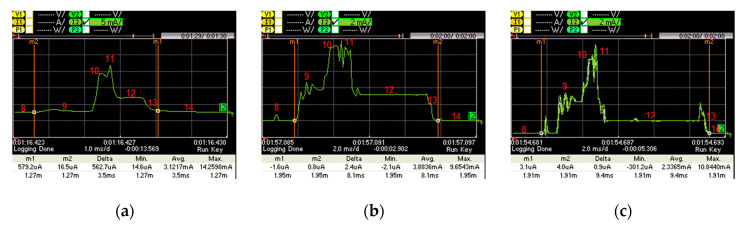
Current consumption of the active part of a connection interval with data packet transmission: (**a**) PCA10028; (**b**) LaunchPad; (**c**) SensorTag.

When there is actual data exchange between two connected devices in our experiment, the IPv6 packets corresponding to states 3 and 4 in Table 1 carry data. Figure 12 depicts the current consumption pattern for such states, when data packet transmission takes place. The characteristics of such states (in terms of duration and current consumption) are further detailed in Table 2. The main differences between Table 1 and Table 2 are that devices in states 10 and 11 consume more energy, compared with states 3 and 4, and state 12 takes longer time than state 5 due to the processing of the received data.

We next calculate the average current consumption of a slave device in a BLE connection with actual data packet transmission (denoted *I_Data-CI_*). This variable can be obtained by using Equation (4):

(4) $$I_{Data-CI}=\left(\overset{14}{\underset{i=8}{\sum }}T_{i}\cdot I_{i}\right)$$

The values of *T_i_* and *I_i_* in (4) represent the state durations and corresponding current consumptions shown in Table 2, except for the sleep time duration (denoted *T_Sleep-WD_*). The latter can be determined based on Equations (5) and (6), where *T_WD-active_* refers to the sum of active time parts within *connInterval* when an IPv6 packet is sent:

(5) $$T_{Sleep-WD\;=\;}T_{CI\;}-\;T_{WD-active}$$

(6) $$T_{WD-active\;=\;}\;\left(\overset{14}{\underset{i=9}{\sum }}T_{i}\right)$$

**Figure 13:**
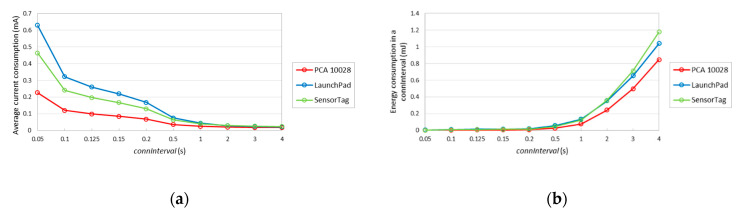
(**a**) Average current consumption for different *connInterval* settings, with data packet transmission; (**b**) energy consumption during a connection interval, with data packet transmission.

The average current consumption of a slave, when a data packet is actually transmitted, for the considered devices, and for different *connInterval* settings, is depicted in Figure 13a. Figure 13b depicts the energy consumption in a connection interval. Similarly, to Figure 11b, for *connInterval* lower than 1 s, LaunchPad consumes more current, while otherwise SensorTag shows a greater current consumption. Curves in Figure 13b are similar to those in Figure 11b, although with higher values. The highest energy consumptions in a connection interval shown in Figure 13b are 1.17 mJ, 1.04 mJ and 0.84 mJ, for SensorTag, LaunchPad and PCA10028, respectively. However, in Figure 11b, these values are 1.07 mJ, 0.90 mJ and 0.81 mJ, respectively.

### 6.3. Current Consumption of the Different Hardware Platforms: A Comparison

**Figure 14:**
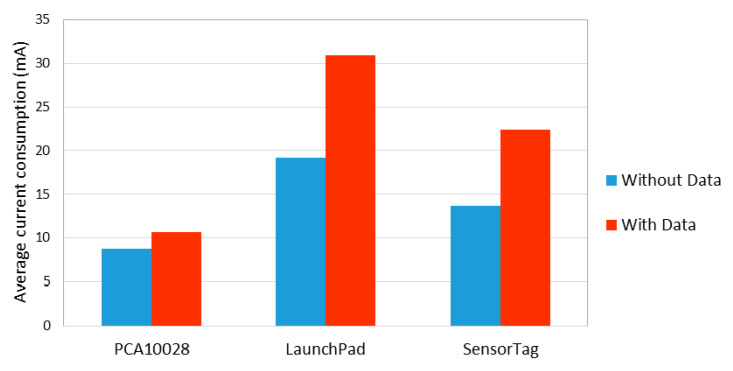
Average current consumption of active parts in connection events with and without data packets, for different device hardware platforms.

Device current consumption during connection intervals with and without data packet transmission were characterized in detail in Section 6.1 and Section 6.2, respectively. Figure 14 compares the average current consumption of the active parts of connection intervals with and without data packet transmission. Note that, during the remaining time, the node is in sleep mode. (Thus, average current consumption during a complete connection interval will vary depending on *connInterval*.) As shown in Figure 14, the average current consumption of the active part of LaunchPad is greater than that of SensorTag and PCA10028. PCA10028 even consumes less current than LaunchPad and SensorTag in absence of data packet transmission. Besides, the current consumption to transmit a data packet with PCA10028 is significantly lower than that of LaunchPad and SensorTag.

### 6.4. Energy Efficiency

In this section, we study the energy efficiency of communication, based on the energy modeling provided in the previous subsection. The energy efficiency per conveyed byte of user data through a BLE connection is calculated by using Equation (7):

(7) $${ℰ}_{Byte}=\;\frac{E_{DataPacket}}{L_{Payload}}$$

where *E_DataPacket_* is the energy that is consumed to deliver one data packet, and *L_Payload_* is the user payload length of that data packet (in our experiments, 18 bytes). *E_DataPacket_* can be obtained by using Equation (8):

(8) $$E_{DataPacket}=I_{Avg}*V*T_{DI}$$

Within a data interval (*T_DI_*), there may be connection intervals without actual data transmission, along with one data connection interval. We take this into consideration in order to compute the average current consumption, denoted *I_Avg_*. The voltage assumed is 3 V, as required by the BLE devices considered in this work. *I_Avg_* can be obtained as shown in Equation (9):

(9) $$I_{Avg}=[\left(1-\frac{T_{CI}}{T_{DI}}\right)*I_{NoData-CI}+\left(\frac{T_{CI}}{T_{DI}}\right)*I_{Data-CI}]$$

where *I_NoData-CI_* and *I_Data-CI_* can be obtained by using Equations (1) and (4), respectively.

**Figure 15:**
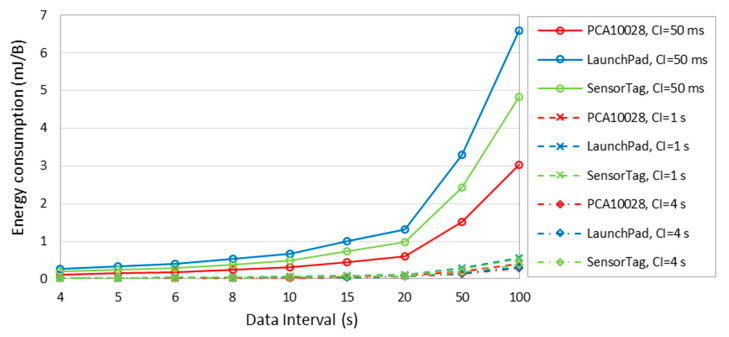
Energy consumption per delivered data byte, for different data intervals, *connInterval* settings, and different BLE hardware platforms.

Figure 15 depicts the energy that is consumed per delivered data byte, for different data intervals, and different *connInterval* settings. The figure shows that the energy consumed to deliver one byte increases with the data interval. That is because, with or without data packet to be sent, connections keep their periodic empty BLE packet transmission, plus the energy consumption in sleep mode intervals, thus connections consume energy while data is not actually transmitted. The worst energy efficiency obtained is 6.59 mJ/B which is found for LaunchPad when the data interval is 100 s and *connInterval* is 50 ms. The best energy efficiency is 11 μJ/B, which is found for PCA10028, when the data interval is 4 s and *connInterval* is 4 s.

### 6.5. Device Lifetime

The device lifetime is an important performance parameter for a battery-operated device, as is typically a 6LN. In addition to the capacity of the energy source, device lifetime is affected by BLE and application-level settings that determine the average current consumption, including *connInterval*, and data interval. In order to determine the device lifetime of our considered hardware platforms, we assume a simple 235 mAh battery. Equation (10) indicates how the 6LN device lifetime, denoted *L*, is computed. *E_Battery_* represents the total energy capacity of the battery, whereas *I_Avg_* denotes the average current consumption of the 6LN. Note that the latter can be obtained as shown in (9). We assume an *E_Battery_* of 235 mAh, which is typical for a button cell battery:

(10) $$L=\;\frac{E_{Battery}}{I_{Avg}}$$

**Figure 16:**
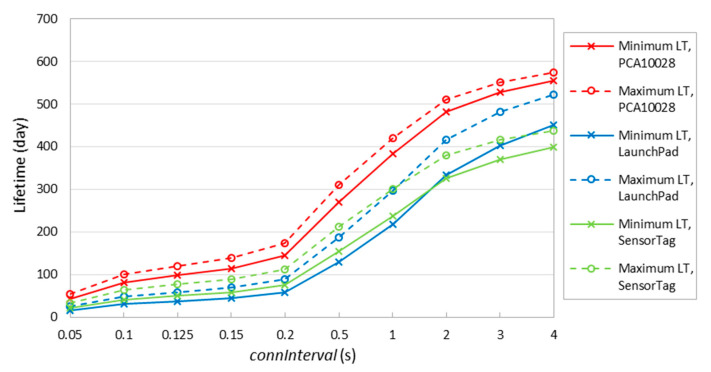
Device lifetime for the different devices considered. Minimum and maximum lifetimes correspond to data interval equal to *connInterval* and infinity, respectively.

Figure 16 shows the theoretical minimum and maximum device lifetime for different *connInterval* settings, and for different devices. In the figure, maximum lifetime curves correspond to the case where there is no data packet payload transmitted in any connection event. Minimum lifetime corresponds to *T_CI_* = *T_DI_* in our study, where we assume that all connection intervals involve the exchange of one packet carrying user data.

Figure 16 confirms that the lifetime of PCA10028 is greater than that of LaunchPad and SensorTag. Moreover, SensorTag lifetime is greater than LaunchPad one when *connInterval* is lower than 1 s, due to the lower sleep mode current consumption of SensorTag discussed earlier. The maximum lifetime is 420 days, 296 days and 300 days for PCA10028, LaunchPad and SensorTag, respectively, when *connInterval* is set to 1 s. The overall maximum lifetime is 573 days, which happens for PCA10028 when *connInterval* is set to 4 s, while the minimum lifetime is ~16 days, which occurs for LaunchPad when *connInterval* is set to 50 ms.

**Figure 17:**
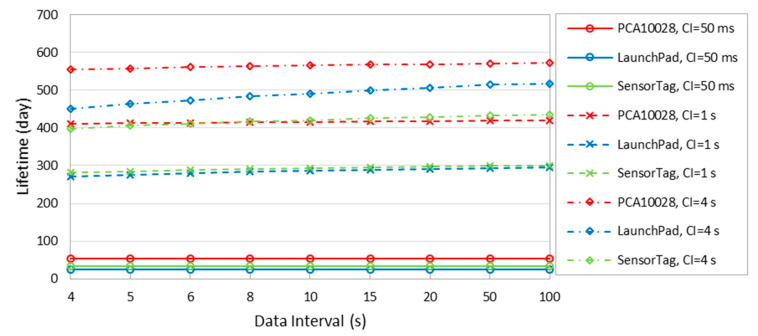
Device lifetime in terms of different *connInterval* and data intervals.

Figure 17 illustrates the device lifetime for different *connInterval* and data interval settings, and for the considered devices. Figure 17 shows that, while lifetime values tend to increase with data interval, the curves are almost horizontal for data intervals significantly greater than *connInterval*. This confirms that the data interval setting has no significant impact on the device lifetime, in contrast with the *connInterval* setting, which is expected to be smaller than or equal to the data interval.

### 6.6. Impact of IPv6 Header Compression

One of the challenges to efficiently support IPv6 in IoT environments is the relatively large IPv6 header (i.e., 40 bytes, by default). Transmission and reception operations consume a significant amount of energy. In addition, a large IPv6 header contributes to fragmentation, as the BLE Link Layer supports a relatively short maximum payload size (of 23 bytes in Bluetooth 4.1). As introduced in Section 2.3, 6BLEMesh supports IPv6 header compression, which mitigates the described issues.

This subsection illustrates the impact of 6BLEMesh IPv6 header compression on current consumption, energy consumption over *connInterval*, and lifetime of a 6LN device. The PCA10028 device hardware platform is assumed for the 6LN.

In our measurements in Section 6.1 and Section 6.2, we used an IPv6 packet payload size of 18 bytes. In its uncompressed form, such IPv6 packet has a size of 58 bytes, which means that a single uncompressed IPv6 packet needs 3 BLE Link Layer data packets to be carried over Bluetooth 4.1. However, when 6BLEMesh header compression is used, the IPv6 packet header is compressed down to 3 bytes, and the compressed packet has a total size of 21 bytes, which fits a single BLE Link Layer data packet. We next quantify the impact of using IPv6 header compression on energy-related performance parameters.

**Figure 18:**
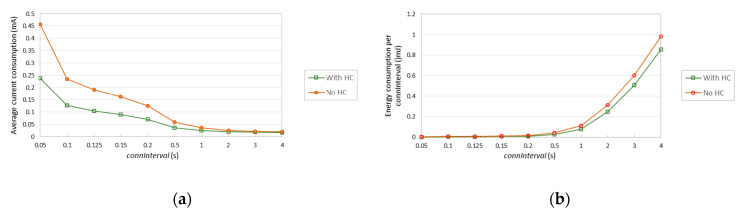
Performance results for a 58-byte IPv6 packet, with and without header compression (HC), for a PCA10028 6LN: (**a**) Average current consumption for different *connInterval* settings; (**b**) energy consumption during a connection interval.

Figure 18a shows the average current consumption of the considered 6LN, assuming *T_CI_* = *T_DI_* for simplicity, when a compressed IPv6 packet of 58 bytes is transmitted with and without IPv6 header compression. Figure 18b illustrates the energy consumption of the 6LN during a *connInterval*, for different *connInterval* settings. The relative impact of header compression increases as *connInterval* decreases, since header compression benefits arise only for transmission intervals (which become more frequent as *connInterval* decreases). For the lowest *connInterval* setting considered (i.e., 0.05 s), header compression reduces the average current consumption by a factor of 1.91. For a *connInterval* of 4 s, header compression decreases the average current consumption by a factor of 1.12.

**Figure 19:**
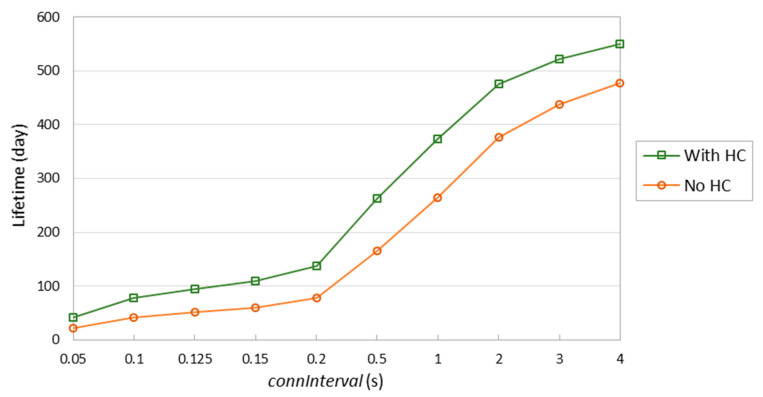
Device lifetime of a battery-operated PCA10028 6LN, for a 58-byte IPv6 data packet, with and without header compression (HC).

Figure 19 shows the lifetime of the considered 6LN device when assuming a battery of 235 mAh and sending a 58-byte IPv6 packet every *connInterval*, with and without header compression. As shown in the figure, header compression allows to extend the device lifetime significantly. Consistently with Figure 18, the relative impact of header compression decreases with *connInterval*. However, even for *connInterval* = 4 s, device lifetime increase (i.e., 73 days) is significant.

## 7. Feasibility of Energy-Constrained 6LRs

In this section, we use the current consumption models derived in the previous section to investigate the feasibility of supporting energy-constrained (e.g., battery-operated) 6LRs in 6BLEMesh. Note that many mesh topology deployments in IoT require intermediate devices to be always on, in order for such devices to be able to receive and forward packets at any time. However, this limits the applicability of such deployments to scenarios where intermediate devices have a virtually unlimited energy source (e.g., the electricity grid).

6BLEMesh might exploit the intrinsic TDMA approach of BLE in order to support energy-constrained 6LRs. A 6LR needs to stay active for the packet exchange with each connected neighbor at the beginning of each connection event. However, a 6LR may remain in sleep mode otherwise.

We next investigate the current consumption, the energy consumption over a *connInterval* time, and the device lifetime of a battery-operated 6LR, by using the energy consumption model derived in the previous section. We consider a Nordic PCA10028 device as the hardware platform for the 6LR, running on a battery of 235 mAh, which is connected to *N* neighbors. We assume an optimized approach where the 6LR communicates with all its neighbors consecutively by, first, waking up (state 9), then participating in the packet exchange with each one of its neighbors (states 10 and 11 for each neighbor), and finally going through postprocessing, radio off and cool down (states 12, 13 and 14) before returning to sleep state, and staying in such state until the beginning of the connection interval. We study how the indicated performance parameters vary as a function of *connInterval*, and of the number of neighbors *N*, and whether actual data packet transmission takes place (assuming an 18-byte data packet payload, and *T_CI_* = *T_DI_*) or not.

**Figure 20:**
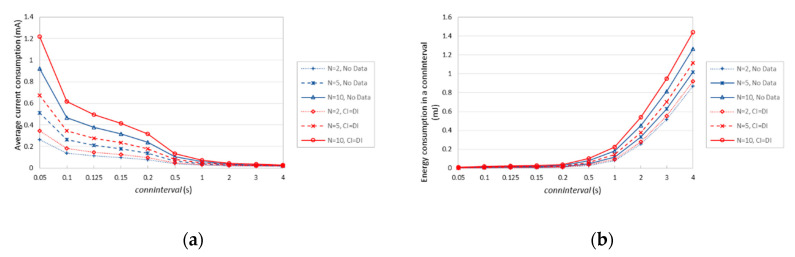
(**a**) Average current consumption of a 6LR, for *N* = 2, *N* = 5, and *N* = 10 connected neighbors. (**b**) Energy consumption of a 6LR in a *connInterval*, for *N* = 2, *N* = 5, and *N* = 10 connected neighbors.

Figure 20a depicts the average current consumption of a 6LR under the described conditions. As expected, the average current consumption decreases with *connInterval*, since sleep intervals become dominant. Relative differences among the considered settings decrease as well. The current consumption difference between sending one data packet every *connInterval* via each connection of the 6LR, and never sending a data packet, increases with the number of neighbors of the 6LR. Figure 20b further illustrates the performance differences among the considered settings in terms of energy consumed in a *connInterval*. For *N* = 10, *connInterval* = 4 s, and *T_CI_* = *T_DI_*, the 6LR consumes roughly 14% more energy over a *connInterval* than in absence of data packet transmission.

**Figure 21:**
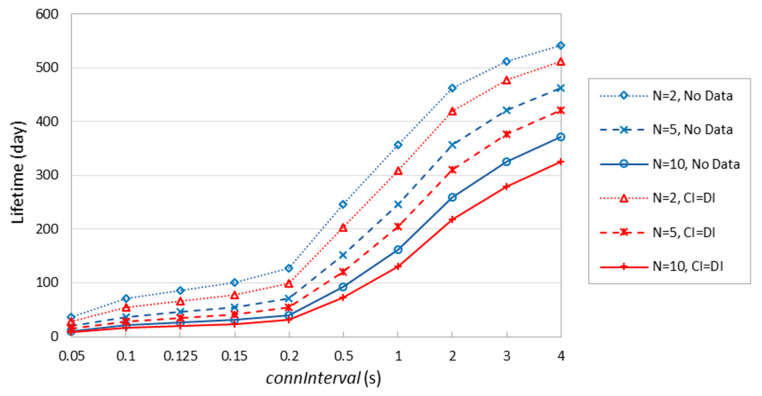
Device lifetime of a battery-operated 6LR, for *N* = 2, *N* = 5, and *N* = 10 connected neighbors.

Figure 21 shows the theoretical lifetime of a battery-operated 6LR device under the considered assumptions. In contrast with Figure 16, the lifetime of a 6LR is significantly reduced, due to the greater amount of activities the 6LR is involved in. It is possible to achieve a battery-operated 6LR lifetime in the order of one year, although that requires high *connInterval* settings (i.e., close to 4 s), or a low number of connected neighbors (e.g., *N* = 2) and *connInterval* ≥ ~1 s. When network node density is high (i.e., *N* = 10), the maximum theoretical lifetime of a battery-operated 6LR takes lies between 326 and 371 days. In some scenarios, it will be feasible to run a 6BLEMesh network comprising energy-constrained 6LRs, especially for high *connInterval*, infrequent data transmission and rather sparse networks.

## 8. Conclusions

In this paper, we experimentally evaluated 6BLEMesh, an IPv6-based solution for BLE mesh networking that is currently being finalized by the IETF. As a side-contribution of the paper, we publicly offer our 6BLEMesh implementation, which comprises the 6LBR, 6LR and 6LN roles. In the evaluation, we focused on latency, RTT, and energy consumption as main performance parameters.

Regarding latency, we showed the different performance characteristics when the sender is the actual source of the packets (approximately, between 0 and *connInterval*), and the case where the sender relays a packet on behalf of other nodes (typically close to *connInterval*). We highlighted the additional delay due to the tick clock, as well as the relationship between the packet sending interval and *connInterval*. RTT measurements are consistent with latency ones.

We modeled the current consumption of the 6LN, for three different BLE hardware platforms. We found significant differences across the considered platforms in terms of current consumption and duration of the different states related to a given action. The models allowed us to determine the device average current consumption, device lifetime, and energy efficiency of communication, as a function of *connInterval* and the application data interval, assuming periodic transmission from a sender. The *connInterval* setting significantly affects the average current consumption, along with the rest of related parameters. However, the impact of the data interval setting on these performance parameters is limited.

The maximum 6LN device lifetime, assuming a simple 235 mAh battery, is 573 days for a Nordic PCA10028 device, whereas it is 519 days for a LaunchPad device, and 436 days for a SensorTag device. We found that IPv6 header compression provides up to 73 days of additional 6LN device lifetime, on a PCA10028 hardware platform.

Furthermore, we investigated the feasibility of a 6BLEMesh network comprising battery-operated 6LRs. Despite the greater amount of tasks that 6LRs are involved in, it is possible to achieve battery-operated 6LR lifetimes in the order of several hundreds of days, which may be sufficient for some use cases and scenarios. Battery-operated 6LR lifetime decreases with the number of 6LR neighbors, and it also decreases as *connInterval* decreases.

## Figures and Tables

**Table 1:** State characterization of a connection interval without data transmission.

State #	Description		Duration (ms)				Current Consumption (mA)		
		Variable	PCA10028	LaunchPad	SensorTag	Variable	PCA10028	LaunchPad	SensorTag
1	Sleep mode	*T_Sleep-ND_*	-	-	-	*I_Sleep-ND_*	0.01	0.01	0.01
2	Wake up	*T_Wake-up_ND_*	1.7	1.6	1.5	*I_Wake-up_ND_*	0.68	2.76	3.39
3	Packet reception from the master	*T_Receive-ND_*	0.20	1.2	0.70	*I_Receive-ND_*	5.90	7.32	6.90
4	Packet response to the master	*T_Response-ND_*	0.29	0.61	0.19	*I_Response-ND_*	10.03	7.37	8.82
5	Post Processing	*T_Post_Proc-ND_*	0.18	0.29	0.50	*I_Post_Proc-ND_*	5.62	3.12	3.30
6	Radio off	*T_Radio_off-ND_*	0.39	0.30	0.26	*I_Radio_off-ND_*	2.83	1.58	1.03
7	Cool down	*T_Cool_Down-ND_*	28.5	0.71	0.18	*I_Cool_Down-ND_*	0.04	0.04	0.02

**Table 2:** State characterization of a connection interval with data packet transmission.

State #	Description		Duration (ms)				Current Consumption (mA)		
		Variable	PCA10028	LaunchPad	SensorTag	Variable	PCA10028	LaunchPad	SensorTag
8	Sleep mode	*T_Sleep-WD_*	-	-	-	*I_Sleep-WD_*	0.015	0.01	0.01
9	Wake up	*T_Wake-up_WD_*	1.7	1.2	2.1	*I_Wake-up_WD_*	0.39	3.0	2.46
10	Packet reception from the master	*T_Receive-WD_*	0.30	1.2	0.72	*I_Receive-WD_*	7.09	6.49	7.04
11	Packet response to the master	*T_Response-WD_*	0.30	0.92	0.67	*I_Response-WD_*	10.76	6.74	4.91
12	Post Processing	*T_Post_Proc-WD_*	0.71	3.9	5.3	*I_Post_Proc-WD_*	4.69	3.12	1.50
13	Radio off	*T_Radio_off-WD_*	0.41	0.67	0.57	*I_Radio_off-WD_*	2.59	1.55	1.59
14	Cool down	*T_Cool_Down-WD_*	23.6	0.61	0.25	*I_Cool_Down-WD_*	0.04	0.04	0.01
